# Antibiofouling Performance by Polyethersulfone Membranes Cast with Oxidized Multiwalled Carbon Nanotubes and Arabic Gum

**DOI:** 10.3390/membranes9020032

**Published:** 2019-02-22

**Authors:** Ahmad Najjar, Souhir Sabri, Rashad Al-Gaashani, Muataz Ali Atieh, Viktor Kochkodan

**Affiliations:** 1 College of Life and Health Sciences, Hamad Bin Khalifa University (HBKU), P.O. Box 34110 Doha, Qatar; 2 Qatar Environment and Energy Research Institute (QEERI), Hamad Bin Khalifa University (HBKU), P.O. Box 34110 Doha, Qatar; ssabri@hbku.edu.qa (S.S.); ralgaashani@hbku.edu.qa (R.A.-G.); mhussien@hbku.edu.qa (M.A.A.)

**Keywords:** oxidized multiwalled carbon nanotubes, arabic gum, polyethersulfone, membranes, ultrafiltration, fouling, biofouling and hydrophilicity

## Abstract

Despite extensive research efforts focusing on tackling membrane biofouling, one of the biggest problems associated with membrane technology, there has been little headway in this area. This study presents novel polyethersulfone (PES) membranes synthesized via a phase inversion method at incremental loadings of functionalized oxidized multiwalled carbon nanotubes (OMWCNT) along with 1 wt. % arabic gum (AG). The synthesized OMWCNT were examined using scanning electron microscopy and transmission electron microscopy for morphological changes compared to the commercially obtained carbon nanotubes. Additionally energy-dispersive X-ray spectroscopy was carried out on the raw and OMWCNT materials, indicating an almost 2-fold increase in oxygen content in the latter sample. The cast PES/OMWCNT membranes were extensively characterized, and underwent a series of performance testing using bovine serum albumin solution for fouling tests and model Gram-positive (*Bacillus subtilis*) and Gram-negative (*Escherichia coli*) bacterial species for anti-biofouling experiments. Results indicated that the composite PES membranes, which incorporated the OMWCNT and AG, possessed significantly stronger hydrophilicity and negative surface charge as evidenced by water contact angle and zeta potential data, respectively, when compared to plain PES membranes. Furthermore atomic force microscopy analysis showed that the PES/OMWCNT membranes exhibited significantly lower surface roughness values. Together, these membrane surface features were held responsible for the anti-adhesive nature of the hybrid membranes seen during biofouling tests. Importantly, the prepared membranes were able to inhibit bacterial colonization upon incubation with both Gram-positive and Gram-negative bacterial suspensions. The PES/OMWCNT membranes also presented more resilient normalized flux values when compared to neat PES and commercial membrane samples during filtration of both bacterial suspensions and real treated sewage effluents. Taken together, the results of this study allude to OMWCNT and AG as promising additives, for incorporation into polymeric membranes to enhance biofouling resistance.

## 1. Introduction

As the human population continues on its exponential growth rate, the global demand for water and wastewater treatments has increased [1]. The development of membrane filtration systems has in turn gained considerable momentum. [2]. Membrane sales worldwide have grown exponentially over the last decade, whilst the average cost of membrane installations have seen a sharp decrease over the same time period [3]. This has been particularly the case with wastewater treatment where the use of membranes to compliment more conventional methods of wastewater treatment has become common place as evidenced by the increasing popularity of membrane bioreactors (MBRs) [4]. These bioreactors offer valuable advantages when compared to activated sludge systems. In comparison, MBRs result in lower levels of sludge production and superior effluent quality, all the while creating a smaller carbon footprint than that produced by activated sludge processes [2,5]. Membrane systems have also proven themselves as the more attractive alternatives to traditional methods of filtration and sedimentation due to the higher-grade filtered water achieved by ultrafiltration (UF).

The promises of membrane technologies are not without their flaws. Of all the problems reported with the use of membranes, fouling is of paramount importance. Fouling can take many forms, including the deposition of organic macromolecules such as proteins, inorganic foulants in the form of metal salts, particulate, and colloidal fouling [6]. Microbial fouling, better known as biofouling has proven to being a major obstacle to membrane-driven systems, particularly in wastewater treatment where biological components are in high abundance [7]. Biofouling is ascribed to any fouling that is caused by microorganisms and their related secretions, namely extracellular polymer substances (EPS) [8]. Unlike other types of fouling, biofouling is the most problematic due to the failure of reducing it during pre-treatment phases since any remnants of microorganisms are capable of multiplying as they self-replicate [9]. This is essentially the reason behind membrane biofouling being labeled as the Achilles heel of membrane technology [10], where it has been a contributing factor to membrane fouling in almost 50% of all cases reported [11].

The process of biofouling is complicated by the many factors and parameters, which influence its initiation, progress, and subsequent development. Microorganisms are transported to a membrane surface by various means—either by fluid dynamic forces or by random Brownian motion—for the non-motile species to more concerted effort by motile species responding to chemotaxic signals from nutrient-laden membrane surfaces [12]. Upon arrival, microbial cells will seek to attach to the membrane surface before establishing a conditioning film composed of EPS. Surface attachment is governed by a number of surface parameters, namely chemical composition, hydrophobicity, surface charge, and roughness [12]. Once attachment has been achieved successfully, a period of cellular growth and metabolic activity follows, enabling the production of EPS by the early colonizers onto the membrane’s surface. The established biofilm reaches a steady state between growth and detachment, the latter caused by shear forces produced by the action of fluids [13]. Detached EPS act as a raft, transporting a host of microbial flora to virgin membrane surfaces, thereby spiraling the development of biofouling on membrane surfaces.

A number of adverse effects result from biofouling on membrane surfaces. Studies have shown that permeate flux strongly correlates with EPS content and mass [14,15], where a decline in membrane flux is observed due to the formation of a mature EPS film, which effectively clogs up surface pores. This leads to increased feed and differential pressure, which are needed to sustain the production rate despite a lower flux. Both the reduced flux and increased operating pressures incur higher energy consumptions, which leads to higher running costs and larger carbon footprint. Permeate quality deteriorates over time as an increase in salt passage occurs due to the build-up of aqueous ions embedded within the EPS film.

A vast array of strategies and technologies have been proposed to tackle the detrimental outcomes caused by biofouling processes. Conventional strategies involve the use of biocidal treatments such as chlorination of feed water by chlorine or chlorine related compounds. The main drawbacks associated with these biocidal agents apart from membrane sensitivity to chlorine, is the production of undesirable by-products of the disinfection process, which find their way into the treated water [6]. By-products such as trihalomethanes and haloacetic acids are suspected cancer-causing chemicals, and hence render the use of chlorine based disinfection unpopular [16].

Ozone’s well known potent oxidizing effects have led to its use as a disinfection tool for wastewater treatment. Despite being effective in deactivating a wide range of microorganisms, ozone’s unstable nature limits it to on-site production. Furthermore, ozone has been implicated in producing bromate in bromide-containing treated waters, a concerning fact since bromate is a known carcinogenic and mutagenic chemical [17].

Ultraviolet (UV) irradiation has seen success in disinfecting wastewater from bacteria and viruses, thereby reducing viable organism count in water. The main problems associated with UV irradiation are cost and its inability in disinfecting and controlling biofouling within membrane modules, thereby limiting its use to small scale automated setups [18].

Biocidal treatments of any kind require flushing and cleaning, both of which incur additional expenses and have to be carried out frequently. Other methods have focused on nutrient limitation, primarily the removal of phosphate from feed water. This can be achieved through the use of coagulants such as lime and alum in chemical precipitation removal methods [19]. The associated maintenance costs and disposal concerns are but a few of the disadvantages of this technique [19,20,21].

The use of antibacterial agents to tackle biofouling has proven problematic due to the realization that the secreted exopolymers (EPS) act as insulating protective shields for the microorganisms encapsulated within, effectively minimizing contact between the microorganisms and antimicrobial agents. On the other hand, the formation and use of low-biofouling membranes have been extensively investigated over the last decade [22,23,24,25]. Efforts have focused mainly on membrane surface modifications, where additives are incorporated into dope solutions in order to improve surface bio-fouling resistance properties, all the while proving to be cost effective and possessing low maintenance features [26]. Most strategies involve enhancing the self-antibacterial properties of the membrane surface by inhibiting biofilm formation in one way or another [25]. Inorganic additives have been widely investigated, ranging from the use of silica and silane nanoparticles [27] to lithium perchlorate [28], to the incorporation of zirconium dioxide [29] and alumina nanoparticles [30] to various polymeric membranes.

Despite widespread research using inorganic materials, the use of organic carbon based additives are increasingly becoming popular in the field of membrane science, particularly where tackling biofouling is the primary focus [31]. Carbon-based materials offer a number of key advantages over the use of inorganic additives, namely their relative ease of production, large scale availability, and perhaps of more topical importance, being more environmentally friendly [31]. Ground breaking discoveries of sp^2^ hybridized carbon derivatives such as graphene, fullerene, and carbon nanotubes have catalysed the research momentum in a vast range of scientific disciplines, including wastewater treatment.

Incorporation of carbon-based additives into polymeric membranes has been reported in several studies, mainly pristine graphene and graphene oxide, both of which have been linked to antimicrobial mechanisms [32,33,34,35]. Ahmed and Rodrigues [34] studied the effect of graphene oxide on wastewater microbial flora. Their work alluded to graphene oxide acting as an antimicrobial agent capable of interfering with bacterial metabolism. Other studies have employed graphene with inorganic additives as composite materials; mainly titanium oxide for photocatalytic treatment of wastewater [36,37] or with iron (III) oxide as adsorbents for water purification [38,39].

More recently, multi-walled carbon nanotubes (MWCNTs) have been incorporated into various polymeric membranes [40,41]. MWCNTs offer ideal transport of water due to their hollow tubular nature, and can possess extremely high surface areas which can be easily modified through the grafting or doping of other functional materials [42]. The major drawback of MWCNTs is the lack of surface functional groups [43]. In one study, polyamide nanocomposites containing carboxylated MWCNTs were fabricated via interfacial polymerization. The synthesized membranes displayed increased hydrophilicity, enhanced membrane flux, and increased salt rejection [40]. Zhu and Wang [41] synthesized ultrafiltration polyethersulfone (PS) membranes modified with zwitterionic MWCNTs. Their composite membranes also displayed enhanced membrane properties including membrane flux and surface hydrophilicity. Whilst their paper briefly studied the antifouling properties of the synthesized membranes using bovine serum albumin (BSA) solution [41], neither study looked at the antibiofouling properties of MWCNT-containing polymeric membranes using bacterial models.

In another recent finding, Arabic gum (AG) as a natural surfactant was implicated as an antimicrobial agent against *Escherichia coli* (*E. coli*) bacteria when blended with polymeric membranes [44,45]. The published work showed that AG acted as a pore-forming agent where its incorporation in both PS and PES membranes led to increased membrane performance and surface properties, similar to the above studies.

In this study, the above encouraging results led to the selection of both MWCNTs and AG as additives to PES as a membrane base polymer, in order to fabricate novel low-biofouling membranes. PES was chosen as a membrane forming polymer owing to its ease of processing, commercial availability, good chemical resistance, and robust mechanical strength [46]. The addition of both AG and oxidized MWCNTs (OMWCNTS) was envisaged to significantly increase membrane hydrophilicity and surface charge, while bolstering membrane flux, in comparison to bare PES membranes. The interaction between both AG and OMWCNT in creating membranes which possess much of the desirable anti-biofouling features was anticipated, in line with previous studies where each of the additives alone brought about some of the hallmarks of smart membranes [41,44]. Specifically, PES/OMWCNTs synthesized membranes would be expected to exhibit robust biofouling resistance when filtering bacterial suspensions and in real treated sewage effluent (TSE) filtration. Importantly, the composite membranes would need to demonstrate anti-adhesive properties against both Gram-positive and Gram-negative bacterial models employed, in order to be considered as robust low-biofouling membranes finding their application in wastewater treatment.

## 2. Materials and Methods

### 2.1. Materials

The following chemicals and analytical grade reagents were purchased: MWCNTs (length ~30 mm and outer diameter ~20 nm) with purity >95%, were obtained from Chengdu Organic Chemicals (Chengdu, China); nitric acid (HNO_3_) (67–69%) from ROMILSpA™ (Cambridge, UK); sulfuric acid (H_2_SO_4_), 94.5% and 30% hydrogen peroxide (H_2_O_2_) from VWR Chemicals® (Radnor, PA, USA); potassium permanganate (KMnO_4_) (≥99%) and N,N-dimethylacetamide (DMA) from Honeywell Fluka®, (Morris Plains, NJ, USA); paraformaldehyde, AG (approximate molecular weight of 250 kDa), bovine serum albumin (BSA; 69 kDa) and hydrochloric acid (HCl) 37% from Sigma-Aldrich^®^ (St. Louis, MO, USA); PES from Solvay, (Brussels, Belgium); m-Endo Agar LES from HACH^®^, (Loveland, CO, USA); mFC agar, phosphate buffered saline (PBS), Rosolic acid and sodium hydroxide (NaOH) all sourced from Fisher Scientific®, (Hampton, NH, USA). All aqueous solutions were prepared in Millipore deionized water (DW). Commercial PM UP150 microfiltration membranes and UE50 PES ultrafiltration membranes were received from Microdyn-Nadir (Wiesbaden, Germany).

### 2.2. Synthesis of OMWCNT

The oxidation and carboxylation of MWCNT was achieved by the following method. MWCNT powder (1 g) was dispersed in a volumetric ratio of 70:30 acid mixtures of H_2_SO_4_ and HNO_3_, respectively, inside a 500-mL beaker surrounded by ice whilst being continuously stirred for uniform dispersion. Next, 6 g of KMnO_4_ were added steadily whilst retaining the temperature below 5 °C. The temperature was then increased and set to 45 ± 5 °C for 2 h using an oil bath. DW (100 mL) was slowly added whilst ensuring the temperature was kept below 20 °C. The beaker was then kept at 85 °C for 1 h under stirring conditions. The experiment was brought to an end via the simultaneous addition of 120 mL of chilled DW and 15 mL H_2_O_2_. The solution was diluted with a small volume of 10% v/v HCl acid solution before being centrifuged at 5000 rpm for 40 min at room temperature. The supernatant was discarded and the process was repeated several times using DW to resuspend the pellet and wash out the acid until pH 7 was reached. The suspension was dried in an oven overnight at 90 °C.

### 2.3. OMWCNT Characterization

The synthesized OMWCNT was characterized by Field emission scanning electron microscopy (FESEM) FEI Versa 3D dual beam from ThermoFisher Scientific, (Waltham, MA, USA) fitted with energy dispersive X-ray spectroscopy (EDX) XFlash 6130 from Bruker, (USA) for morphological and elemental analysis of the synthesized MWCNT samples. Transmission electron microscopy (TEM) using FEI Talos F200X, ThermoFisher Scientific, (Billerica, MA, USA) was carried out on a small sample of OMWCNT suspension, which was allowed to dry on to a perforated carbon coated grid before imaging.

Thermo-gravimetric analysis (TGA) for thermal stability and indirect analysis of adding carboxylic acid and other oxygen-containing functional groups was performed using a TGA 55, TA Instruments (New Castle, DE, USA). The samples were heated up to 800 °C at a ramp rate of 10 °C/min under nitrogen atmosphere.

### 2.4. Membrane Synthesis and Casting

Casting solutions were prepared using 16 wt. % PES polymer dissolved in N,N-dimethylacetamide (DMA) solvent. Dope solutions were aliquoted, and 1 wt. % AG and different loadings of OMWCNT were added to cast the nine membranes detailed in Table 1. The casting solutions were subject to a period of 1 h sonication using a Q500 sonicator probe (Thomas Scientific, Swedesboro, NJ, USA) whilst being stirred using a labForce digital hotplate stirrer (Thomas Scientific, Swedesboro, NJ, USA) to allow for effective dispersion of the nanofiller throughout the solution. The solution was then degassed for 1 h in order to remove air bubbles. The membranes were cast according to standard phase inversion techniques using a Labcoat Master casting system (PHILOS, Gyeonggi-do, Korea) onto a glass plate. The casting speed was set at 3 m/min with a knife gap height of 200 µm. The glass was immersed into a coagulation bath filled with DW where the solvent (DMA)/non-solvent (DW) exchange took place causing the precipitation of the polymeric membrane onto the glass. The membranes were washed thoroughly with DW in order to remove any residual solvent before being stored in DW.

### 2.5. Membrane Characterization

#### 2.5.1. Membrane Surface Morphology, Pore Size and Total Porosity

Field emission scanning electron microscopy (FESEM) (with set vacuum conditions at 3 kV) was utilized to study the surface and cross-sectional morphologies of the synthesized membranes. Liquid nitrogen was used in the preparation of the cross-section samples whilst all samples were sputtered with gold coating (2.5 nm thickness).

The gravimetric method for assessing membrane porosity was used [47]. This entailed cutting triplicate square samples of each membrane to a set measured size before weighing the membranes whilst wet, ensuring any excess DW was first wiped off before incubating them overnight at 45 °C. The membranes were then re-weighed the next day in order to obtain the dry mass. The average total porosity was calculated using Equation (1) below:

(1) $$\varepsilon \;\left(\%\right)=\;\frac{w_{w}-w_{d}}{A\;\times l\;\times \;\rho }\;\times 100\%$$

where *w_w_* and *w_d_* represent the wet and dry masses of the membrane sample, respectively, *ρ* represents the density of DW at 25 °C water (998 kg/m^3^), *A* is the effective surface area of the membrane sample (m^2^), and *l* is the membrane thickness (m).

The average pore size for each membrane was derived by applying the Guerout–Elford–Ferry equation below:

(2) $$r_{m}=\sqrt{\frac{\left(2.9-1.75\varepsilon \right)8\;\eta \;l\;Q}{\varepsilon \;A\;\Delta P}}$$

where ε is the total porosity, *η* is the viscosity of DW at 25 °C (8.9 × 10^−4^ Pa·s), *Q* is the volume of DW permeate (m^3^/s), and Δ*P* is the operating pressure (typically 1 bar).

#### 2.5.2. Surface Roughness

An atomic force microscope (AFM) (Bruker’s Dimension Icon, Billerica, MA, USA) was employed for analyzing the surface roughness of membrane samples. To that end, triplicate samples were glued onto glass slides before scanning. The membrane samples were scanned using multiple scan sizes to generate height maps of the membrane samples.

#### 2.5.3. Membrane Hydrophilicity and Surface Charge

Water contact angle measurements were obtained using a KRÜSS DCA-25 Drop Shape Analyzer (KRÜSS GmbH, Hamburg, Germany) in order to evaluate the hydrophilicity and wettability of the synthesized membranes. The volume of each DW droplet was set at 2 µL and five different readings were taken at random locations on each membrane sample in order to calculate an average set of water contact angle values. Angles were measured using sessile drop orientation with a Young Laplace fitting method at room temperature.

The membrane surface charge was evaluated via the streaming potential method in a SurPASS™ 3 electrokinetic solid surface analyzer (Anton Paar, Graz, Austria). Triplicate samples for each membrane were cut to size and fixed onto a sample cell holder, maintaining a 100 µm gap between the two membrane samples. KCl solution (1 mM) was passed through the measuring cell and zeta potential values were obtained for a range of pH values at room temperature using buffers (0.1 M NaOH and 0.1 M HCl) to alter streaming salt solution pH.

#### 2.5.4. Mechanical Testing of Membranes

Dynamic mechanical analysis (DMA) tests were performed using a Q 800 DMA (TA Instruments, New Castle, DE, USA) which uses a motor generated force on the sample whilst displacement sensors measure strain, force, and amplitude. A uniaxial tensile load at a ramp displacement rate of 150 µm/min was configured. Triplicate membrane samples were initially dried before being cut into rectangular shapes with approx. dimensions of 22 mm × 6.5 mm. The thickness of each membrane sample was measured using a digital micrometer gauge. Young’s modulus for each membrane sample was derived from the initial gradient of the generated strain (%)–stress (MPa) graphs. Average values were recorded and used to evaluate the mechanical properties of each membrane.

#### 2.5.5. Thermal Stability Characterization

TGA of the membranes was carried out by heating the membrane samples at a ramp rate of 10 °C/min up to 650 °C under nitrogen atmosphere using a TGA 55 device (TA Instruments, New Castle, DE, USA). The derivative thermo-gravimetric analysis (DTGA) weight percent per temperature for each sample was used to infer the relative percentage mass of water held in the membranes and oxygen content in the membrane material (see Figure S1 for results).

### 2.6. Membrane Performance Testing

A pressurized dead-end stirred cell (HP4750X from Sterlitech, Kent, WA, USA) was employed for the filtration tests. The initial flux of the membrane samples was determined using DW at a set pressure (typically 1 bar). The permeate flux (J) was determined using Equation (3) below:

(3) $$J=\frac{Q}{A\;\times \;T}$$

where *Q* is the permeate volume collected in time (*T*), and *A* represents the effective cross-sectional area (m^2^) of the membrane in the filtration cell.

#### 2.6.1. Antifouling Tests

Membrane resistance to organic fouling was evaluated via filtration of 0.1 wt. % BSA solutions. The initial DW flux (*J*_*w*1_) and final DW flux (after BSA filtration) (*J*_*w*2_) were determined. The normalized flux calculated as a ratio of *J*_*w*1_/_*w*2_, served as an indicator of DW flux recovery. The total fouling rate (*R_t_*), reversible (*R_r_*), and irreversible (*R_ir_*) fouling rates were also calculated from the normalized flux data using the Equations (4)–(6), respectively.

(4) $$R_{t}=R_{r}+R_{ir}$$

(5) $$R_{r}\;\left(\%\right)=\left(\frac{J_{w2}-J_{BSA}}{J_{w1}}\right)\times 100\%$$

(6) $$R_{ir}=\left(\frac{J_{w1}-J_{w2}}{J_{W1}}\right)\times 100\%$$

where *J*_*w*1_ is the DW flux before BSA filtration, *J_BSA_* is the flux for BSA solution, and *J*_*w*2_ is the DW flux after BSA filtration.

BSA rejection (*R*) was calculated using Equation (7). A Shimadzu spectrophotometer (Japan) calibrated at 258 nm wavelength was used to read the optical density (OD) of permeate and feed solutions.

(7) $$R\;\left(\%\right)=\left(1-\frac{C_{p}}{C_{f}}\right)\times 100\%$$

where *C_p_* and *C_f_* are the concentration of BSA in the permeate and in feed solution, respectively.

#### 2.6.2. Anti-Biofouling Properties of the Fabricated Membranes

The anti-biofouling properties of the fabricated membranes were studied with model suspensions of both Gram-positive and Gram-negative bacteria (*E. coli* and *Bacillus subtilis* (*B. subtilis*)) suspensions, respectively, grown to 0.025 optical density (OD) measured at 600 nm. Membrane samples were placed in bacterial suspensions for 10 min before being incubated on agar plates overnight at 37 °C. The next day the samples were fixed with 4% paraformaldehyde before being washed with PBS. The membranes were sputtered with gold coating before SEM imaging.

Biofouling resistance of synthesized membranes was also tested after contact with bacterial suspensions over a duration of one, three, and seven days. To that end, membranes were incubated with *E. coli* suspension at the stated times, after which time the flux was measured and compared with initial flux prior to incubation. Such experiments were also repeated with real TSE in place of bacterial suspension.

In a different experiment, the membranes were tested for their biofouling resistance upon having *E. coli* suspension (0.025 OD) pipetted on the membranes’ active surface and left incubated overnight at 37 °C. The membranes’ normalized flux was determined post incubation and compared with initial DW flux for each sample. For comparison purposes, two commercial membranes, namely PM UP150 and UE50 PES (Microdyn-Nadir, Wiesbaden, Germany) were tested.

BSA rejection for fouled membranes was determined for a selection of both fabricated PES/OMWCNT and commercial membranes, in order to compare the rejection performance of the membranes before and after filtration with *E. coli* suspension (0.025 OD).

#### 2.6.3. Membrane Performance during Filtration of Real TSE

The developed membranes were subjected to 90 min of real TSE filtration. For comparison, two commercial PES membranes, namely PM UP150 and UE50 membranes were also tested with TSE in the same experimental setup. This experiment was repeated with filtration of bacterial suspension instead of real TSE for the same duration.

To assess bacterial content in the membrane permeate, the permeate samples derived from TSE filtration were passed through a 0.22 µm cellulose Whatman filter disc (Sigma-Aldrich, St. Louis, MO, USA). The discs were incubated overnight on either LB agar (for total bacterial count) at 37 °C, or endo agar (selective media for total coliform bacteria) or m-Fc agar with 1% Rosolic acid (selective media for fecal coliforms in water).

## 3. Results and Discussion

### 3.1. Characterization of MWCNT and OMWCNT

**Figure 1:**
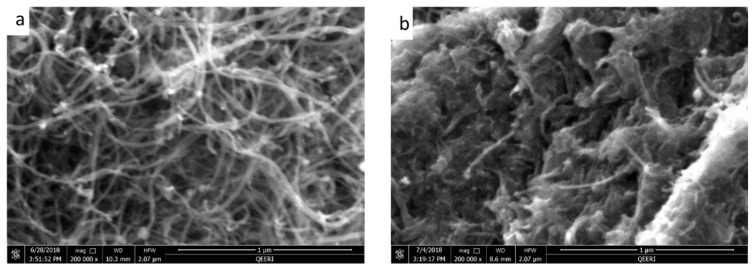
Field emission scanning electron microscopy (FESEM) images for multiwalled carbon nanotubes (MWCNT) (**a**), oxidized multiwalled carbon nanotubes (OMWCNT) (**b**).

The oxidative effects on raw MWCNT can be seen clearly from the Field emission scanning electron microscopy (FESEM) and energy dispersive X-ray spectroscopy (EDX) data shown in Figure 1. Whilst the FESEM images do not reveal substantial morphological differences between the synthesized OMWCNT and MWCNT used as a starting material (Figure 1a,b), EDX data shows the successful oxidation of the MWCNT. EDX data shows a 1.9 fold increase in oxygen content in the product (OMWCNT) compared to that in the starting material (MWCNT; Table 2 and Table 3; see S1 for EDX mapping graphs).

**Figure 2:**
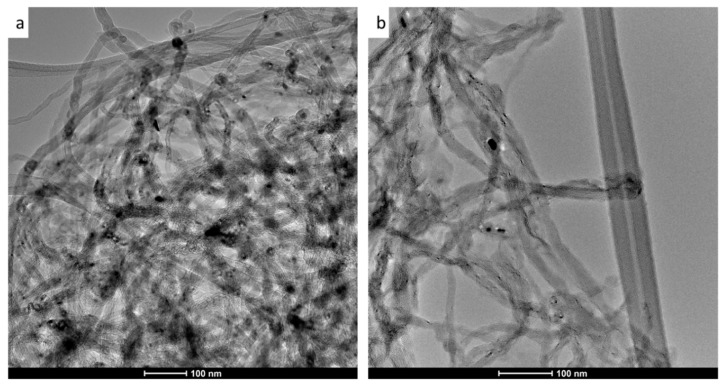
Transmission electron microscopy (TEM) images of multiwalled carbon nanotubes (MWCNT) (**a**) and oxidized multiwalled carbon nanotubes (OMWCNT) (**b**).

TEM images in Figure 2 reveal no significant morphological change to the nanotubes before and after acid treatment. Both images show that the MWCNT exist as bundles with distinct long tubular structures, in line with other studies which presented TEM images with similar findings [40,48]. Importantly, the image in Figure 2b displays tubes with smooth surfaces with no apparent defects that might have arisen due to the oxidation process.

**Figure 3:**
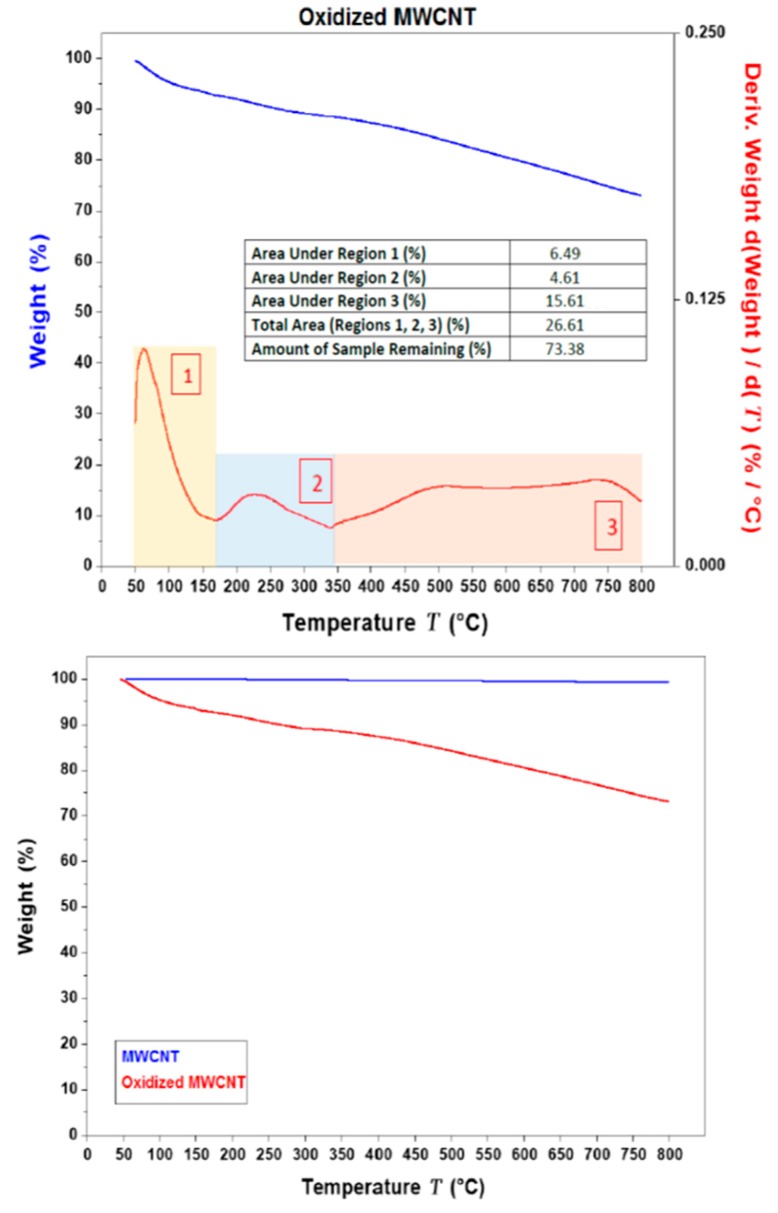
(**a**) Thermo-gravimetric analysis (TGA) (in blue) and derivative thermo-gravimetric analysis (DTGA) (in red) thermograms for OMWCNT. The area under the different segments of the graph are quantified in the insert. (**b**) Overlay of the TGA thermograms for both MWCNT (in blue) and OMWCNT (in red).

The TGA (and DTGA) thermograms for MWCNT and OMWCNT samples are shown in Figure 3. The TGA thermogram overlay for MWCNT and OMWCNT seen in Figure 3b emphasizes the difference in the two material’s thermal stability. The thermogram for MWCNT is almost constant throughout the temperature range. The stark difference in thermograms can be attributed to the successful oxidation of OMWCNT sample, where both the introduction of oxygen functional groups and adsorption of water molecules explain the staggered decomposition of the OMWCNT sample. An initial weight loss is observed just below 100 °C, which accounts for the loss of water molecules adsorbed by the oxidized carbon nanotubes (see area 1 in Figure 3a). A further gradual weight loss continues until 350 °C, which was accounted for by the formation and subsequent loss of carbon dioxide gas (see area 2 in Figure 3a). The 5% derivative weight can therefore be attributed to the oxygen-containing functional groups. The remaining weight of the sample nearing 73% accounts for the non-combusted material or ash (area 3 in Figure 3a). Importantly, the TGA curve for OMWCNT remains stable up to 100 °C, which confirms its thermal stability, thus enabling OMWCNT to be used in a wide range of membrane applications.

### 3.2. Membrane Characterization

**Figure 4:**
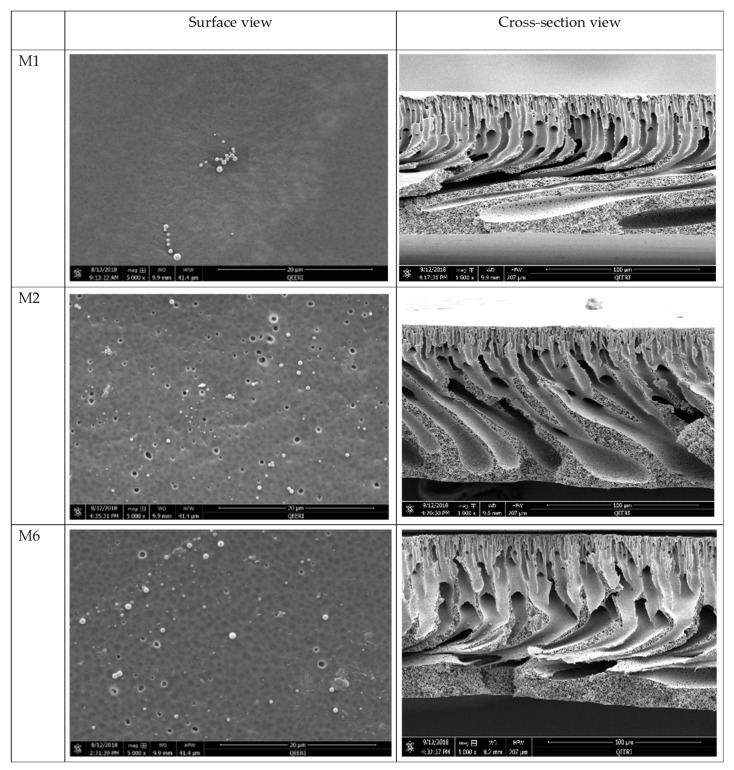
FESEM surface and cross-section images of selected synthesized membranes (M1, M2, and M6).

The Field emission scanning electron microscopy (FESEM) images reveal no radical visible changes in surface morphology upon addition of AG (M2) or with the addition of AG with OMWCNT (M6) when compared to M1 surface samples (Figure 4). This observation is in line with other studies which reported similar findings where incorporation of AG [44] or MWCNTs [41] did not alter the overall surface morphology, nor did it cause any disturbance to the surface structural integrity. Interestingly, an increase in surface porosity can be seen upon incorporation of AG and OMWCNT (M2 and M6, respectively, in Figure 4), compared to the neat membrane (M1).

The cross-sectional images reveal more prominent changes when OMWCNT and AG were incorporated into the polymeric membranes (Figure 4). Unlike M1 (bare PES membrane), M2 and M6 membrane samples had different structural features not seen in neat PES membranes. Despite all samples displaying a typical asymmetric structure where the top surface layer is denser than the finger-like channels in the lower matrix of the membrane structure, development of the channel structures was clearly visible in samples M2 and M6, which incorporated AG or both AG and OMWCNT in their respective casting solutions. Zhu and Wang [41] reported a similar trend where incremental loadings of MWCNTs led to enhanced finger-like pore structures.

The asymmetric structure of the membranes, which is caused by the instantaneous demixing between solvent and DW in the coagulation bath during the phase inversion membrane preparation process, was significantly more pronounced in the membrane samples containing AG and OMWCNT compared to neat PES membrane samples (Figure 4). Such enhancement can be attributed to the introduction of hydrophilic additives, namely AG and OMWCNT. The hydrophilic nature of these two additives together acts to speed up the demixing process by increasing the thermodynamic instability in the system [47]. Larger macrovoids in the walls of the finger-like projections are evident in the composite membranes. These macrovoids and the developed finger-like structures enhance the permeation of water, thereby allowing for increased membrane flux [49].

**Figure 5:**
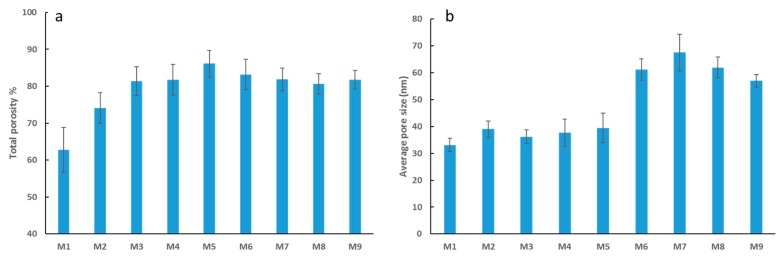
Total porosity of fabricated PES membranes (**a**), and average pore size of fabricated PES membranes (**b**). Each bar represents the mean ± SD (standard deviation) of three independent readings.

Support for increased transport of water for the composite membranes compared to neat PES membranes is provided in Figure 5, which displays both the average total porosity and the average pore size for each membrane. In general, the more abundant and larger the pores are, the lower the water permeation resistance [50]. Figure 5a shows total porosity of PES membranes increasing with incremental loading of OMWCNT up to M5 (0.05 wt. % OMWCNT), after which the total porosity decreases slightly. Although no significant differences are observed between M6 to M8 (note the error bars), a similar phenomenon was reported by Zhu and Wang [41] where they observed a decrease in total porosity when the loading of MWCNTs was greater than 0.2 wt. %. At higher nanofiller loadings, OMWCNTs have a tendency to aggregate into bundles, resulting in some of the pores being clogged, thus accounting for the slight drop in total porosity at elevated OMWCNT loading [41,51]. Interestingly, the addition of AG alone to the polymer membrane is seen to increase the total porosity by 18%.

The addition of both AG and OMWCNT brings about a significant increase in pore size (Figure 5b) with M7 (3 wt. % OMWCNT) having the largest determined pores sized (approx. 100% increase from M1 pore size). The higher porosity can be accredited to the presence of hydrophilic AG and OMWCNT additives, which act as non-solvent agents, thereby accelerating the demixing process. Such rapid demixing results in more porous structures with notably larger pore sizes [44,45]. Interestingly, the addition of AG alone increases the porosity by 18% when compared with neat PES samples. Conversely, M9 samples, which lack AG but contain 0.3 wt. % OMWCNT, retained relatively large pore sizes. These results implicate OMWCNT as being the major contributor to the increase in pore size, despite AG having an additive albeit minor effect. This conclusion is supported by work done by Manawi et al. [44], where the authors noted negligible differences in pore size when membranes were cast with 1 wt. % AG or bare PES. Another study by Zhu and Wang [41] found that adding MWCNTs to their fabricated membranes leads to an overall increase in pore size. The authors attributed this increase in pore diameter to the hydrophilic nature of the MWCNT additives which encourage instantaneous phase separation during the membrane fabrication process [41].

The subtle decrease in pore size calculated for M8 (Figure 5b) which had the highest OMWCNT loading correlates with previous studies which indicated that beyond a certain loading threshold; the casting solution becomes too viscous causing a delayed demixing process between the solvent and non-solvent in the coagulation bath during membrane casting [41,51].

**Figure 6:**
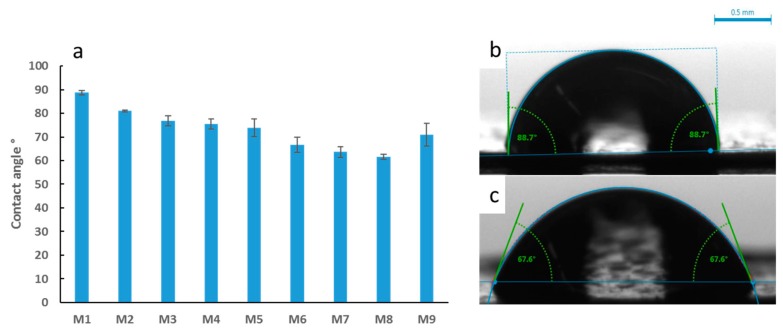
Average water contact angle readings for the synthesized PES membranes (**a**), sample photographs of water droplets with angle measurements for selected samples of M1 (**b**) and M8 (**c**). Each bar represents the mean ± SD of five independent readings.

Figure 6a depicts the results for the average water contact angle values determined for the synthesized PES membranes, whilst Figure 6b,c shows the shapes of the water drops on different membrane samples. Smaller contact angle measurements indicate increased wettability and hence more hydrophilic membrane surfaces. The overall trend shows a decrease in the contact angle by 31% as OMWCNT content increased from 0 wt. % in M1 to 5 wt. % in M8. The 9% fall in contact angle from M1 to M2 samples can be attributed to the introduction of AG into the polymer membrane. This agrees with a previous study involving the addition of AG, where the authors attributed the decrease in contact angle to the charged polysaccharide residues present in AG, which effectively hydrophilized the membrane surface [45]. The synergistic hydrophilization of PES membranes by both AG and OMWCNT was deduced from the significantly lower water contact angle readings obtained for M3 through to M8, whose membrane samples contain both additives. M9 samples buck the trend with a noticeably larger contact angle. The decreased hydrophilicity exhibited by these samples could be qualified by the lack of AG at the membrane’s surface, meanwhile implicating OMWCNT alone to account for the decrease in contact angle relative to the neat PES membrane.

One of the fundamental hallmarks of low-biofouling membranes is surface charge [52]. It has been well documented that a net negative charge on membrane surfaces aids in deterring bacterial attachment, and hence biofilm formation due to repulsive forces acting against the negatively charged bacterial cell walls [6,26,33]. Net negative charges on different bacterial species have been attributed to the presence of teichoic acid residues (which are negatively charged due to the abundance of phosphate groups) associated to the peptidoglycan cell wall of Gram-positive bacteria [53]. Gram-negative bacterial species confer an overall negative charge due to the phospholipid and lipopolysaccharide macromolecules covalently bonded to their outer cell membrane [54].

**Figure 7:**
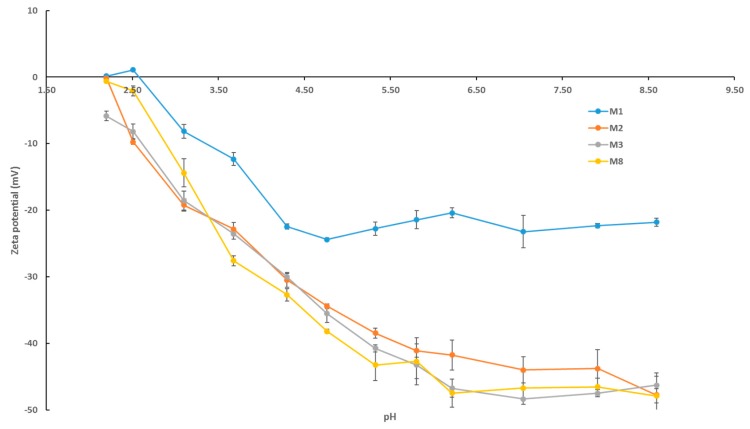
pH scans for selected fabricated PES membranes displaying zeta potential readings. Each bar represents the mean ± SD of three independent readings.

The zeta potential data presented in Figure 7 for selected fabricated membranes reveals a number of important trends. Firstly, we notice that all membrane samples increase in negative charge as the pH increases from acidic to basic conditions. This trend is a result of the selective adsorption of the negatively charged chlorine ions on the membrane surface [55]. Secondly, it is observed that the neat PES membrane (M1) possesses the smallest negative charge across all pH values. The surface negative charge of membranes with AG alone (M2) and with AG/OMWCNTs (M3 and M8) is increased, with M8 having the greatest negative charge across most of the pH range. At neutral pH for instance, the negative zeta potential sees a 100% increase for M8 surfaces compared to M1 samples. The increase in negative surface charge for the composite membranes in comparison to the neat PES membrane samples can be explained by the presence of hydrophilic residues of the AG [45], which contain carboxylic groups as well as the hydrophilic functional groups present on the OMWCNTs [40,41].

**Figure 8:**
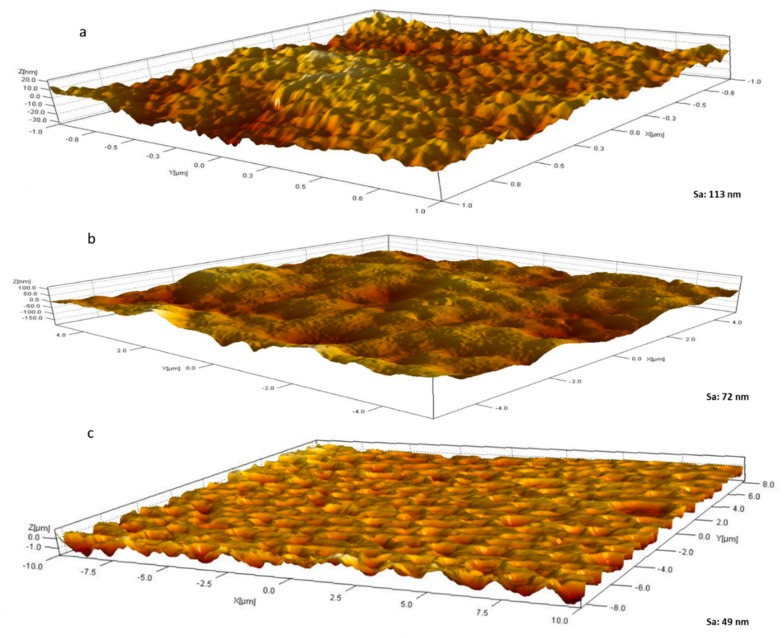
3D AFM surface images for M1 (**a**); M7 (**b**); and M8 (**c**) samples. Mean surface roughness (Sa) values are indicated.

Another important feature of biofouling resistant membranes is membrane roughness [52]. In principle, the rougher the membrane surfaces, the more ‘nooks and crannies’ present which serve as microhabitats for microbial communities to flourish and establish their biofilm. On the other hand, membranes with lower surface roughness and surface energy provide more robust antibiofouling properties and membrane performance [56,57]. Figure 8 shows three-dimensional (3D) AFM images for M1 (neat PES) and composite membranes M7 and M8 samples. The bright areas represent the highest points or peaks of the membrane surface, whilst the darker areas indicate valleys and lower heights. AFM images reveal a stark difference in surface roughness, with neat PES possessing a mean surface roughness more than double that of M8 that had the highest OMWCNT loading (Sa values of 113 nm and 49 nm, respectively). These findings are in line with other research where neat membranes were reported as having significantly higher mean roughness values compared to composite or hybrid membranes ([47,56,57,58]. These results indicate that the addition of nanofillers was compatible with the membrane matrix and did not cause high electrostatic interaction between the two [59]. Furthermore, these results indicate that the composite PES membranes incorporating OMWCNT possess smoother surfaces, further assisting their anti-biofouling capabilities.

**Figure 9:**
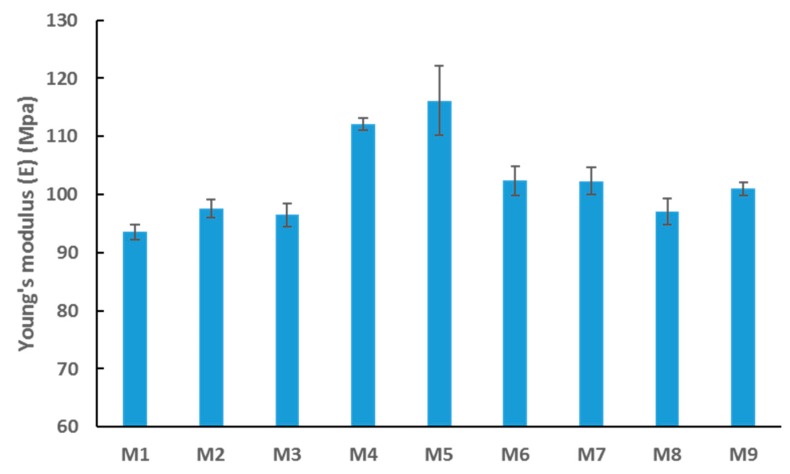
Average Young’s modulus values for synthesized PES membranes. Each bar represents the mean ± SD of three independent readings.

The mechanical properties of the synthesized membranes were tested using the DMA technique. Figure 9 reveals an interesting relationship between incremental loading of nanofiller (OMWCNT) and membrane stiffness (inferred from Young’s modulus values). In general, the stiffer the material, the higher the modulus, implying a less stretchy material. The results indicate that the stiffness increased up to a certain loading of OMWCNT (0.5 wt. %) and then decreased at higher loadings (M6 to M8). A slight increase in stiffness was seen for M9 samples where the nanofiller was significantly less (0.3 wt. %) and lacked the incorporation of AG. As already discussed, agglomeration of OMWCNT in the matrix is likely to occur at higher loadings. Owing to this phenomenon and the increased pore sizes of M6, M7, and M8 samples (Figure 5b), the overall integrity of the membranes’ mechanical strength could well be affected [60,61]. It is worth noting that the observed decrease in Young’s modulus does not appear significantly large. Importantly, the decline in modulus did not reach baseline stiffness levels (Young’s modulus for M1 samples).

### 3.3. Performance Testing of Synthesized Membranes

**Figure 10:**
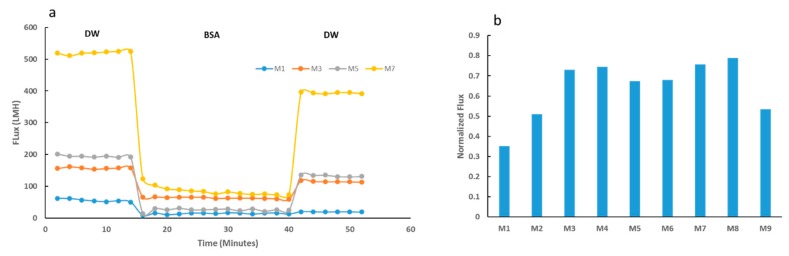
Fouling tests using BSA solution for selected membranes. (**a**) The initial deionized water (DW) flux was measured before bovine serum albumin (BSA) filtration and then final DW flux was measured for each selected membrane. (**b**) Normalized flux data for selected PES membranes, calculated as a ratio of final DW flux to initial DW flux.

Results for the fouling tests modeled with BSA solutions are shown in Figure 10. Figure 10a indicates the membrane flux for selected membranes at different stages of the fouling test. M7 sample showed a superior initial DW flux, with a 9-fold increase from the neat membrane’s initial flux with DW. Such tremendous improvement in flux was expected as it is well accepted that membranes with higher hydrophilicity, total porosity and average pore size possess higher flux [47]. Upon BSA filtration, all membranes indicated a decline in membrane flux. Importantly M7 samples showed much higher flux recovery compared to the neat membrane as supported by the normalized flux data shown in Figure 10b. The normalized flux data highlights the varied recovery competencies for all nine synthesized membranes. M7 had a normalized flux, which was comfortably over 100% greater than that of the neat PES M1 sample. The enhanced flux attributed to the increased hydrophilicity and surface charge explains the improved flux recovery performance exhibited by the composite membranes.

**Figure 11:**
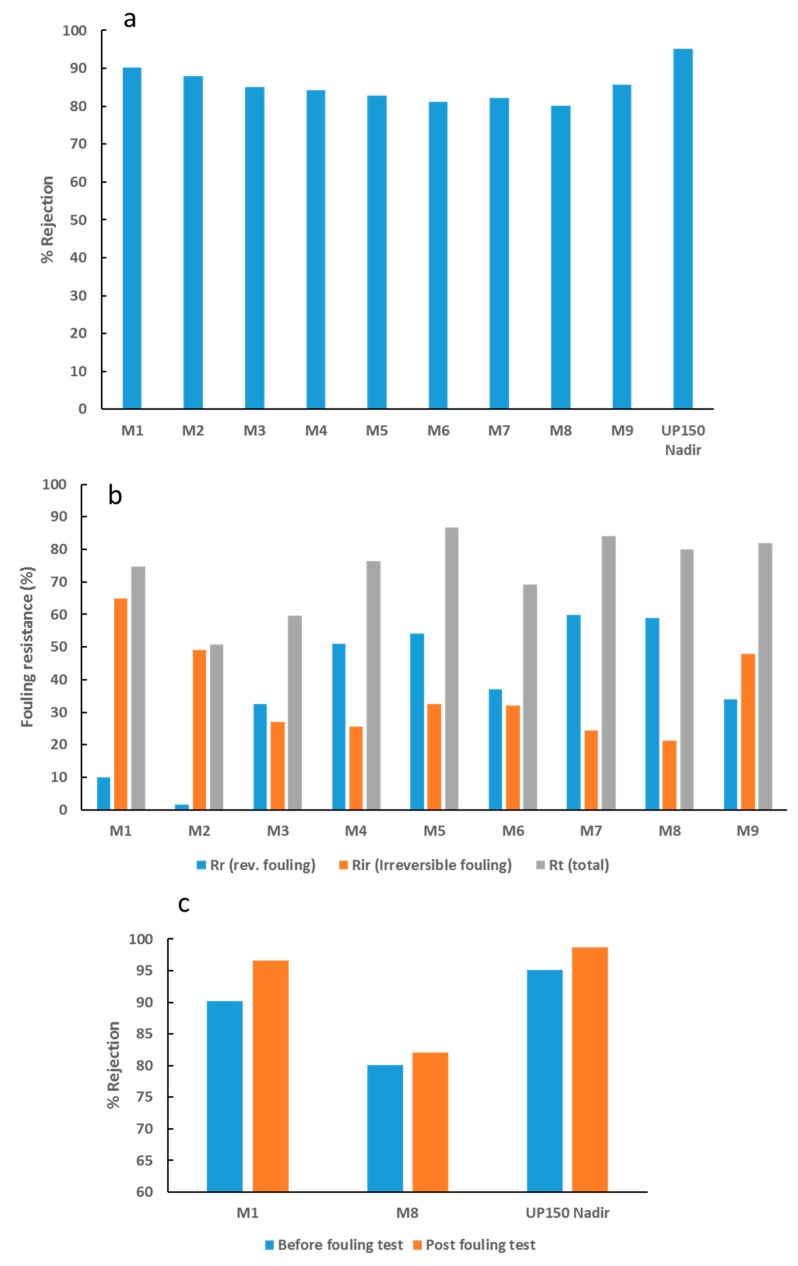
Rejection and fouling data for fabricated PES membranes. (**a**) BSA rejection values, (**b**) different types of membranes fouling derived from BSA solution filtration tests, and (**c**) BSA rejection values for M1, M8, and UP150 Nadir commercial membrane before and after filtration with *E. coli* suspension.

Figure 11a shows a decline in BSA rejection as the loading of OMWCNT increases. The observed decrease can be accounted for by the increase in pore size as the nanofiller content increases (Figure 5b). The decreased rejection as a trade-off to increased pore size has been widely reported in the literature [45,58,62]. Interestingly, pore size seemed to play a more important role than membrane hydrophilicity and surface charge in terms of rejection performance. Similar findings have been reported in other studies, where hydrophilic nanofillers were incorporated into polymeric membranes [41,58]. Figure 11b displays data for a range of fouling resistance parameters. Importantly, a significant decline in irreversible membrane fouling was observed as the wt. % of OMWCNT loading increased. The increase in membrane surface hydrophilization, coupled with the increased negative surface charge associated with the synthesized composite membranes can account for the observed trend. Since BSA molecules exhibit a negative charge at neutral pH due to their low isoelectric point [63], electrostatic repulsive forces limit the absorption of BSA on the membrane surface and pore structures. Ultimately, this leads to a decrease in irreversible fouling as seen in Figure 11b.

**Figure 12:**
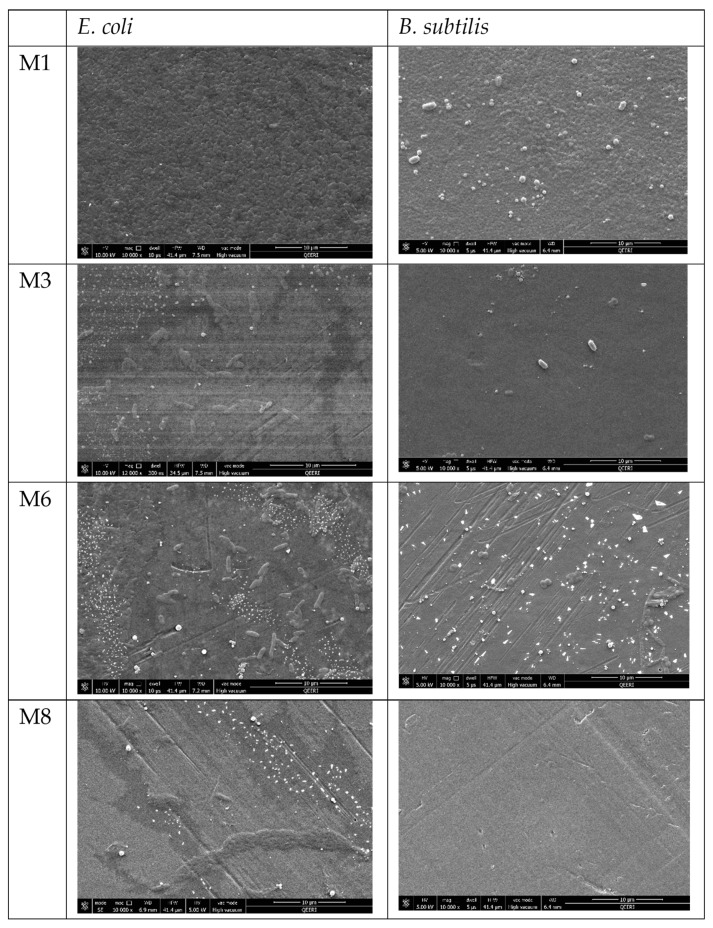
FESEM images for selected PES membrane surfaces after incubation with *E. coli* suspension (**left side panel**) and with *B. subtilis* suspension (**right side panel**).

**Figure 13:**
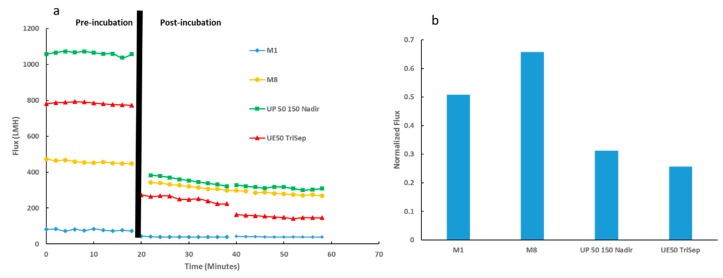
Membrane performance testing for PES-synthesized membranes and commercial membranes. (**a**) Flux readings for membranes before and after incubation with *E. coli* suspension (denoted by black line). (**b**) Normalized flux data for membranes tested.

**Figure 14:**
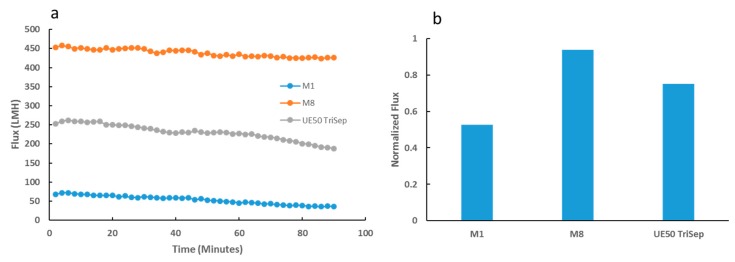
Flux readings for synthesized and UE50 TriSep membranes over a prolonged period of bacterial suspension filtration (**a**). Normalized flux data for each of the three membrane samples (**b**).

**Figure 15:**
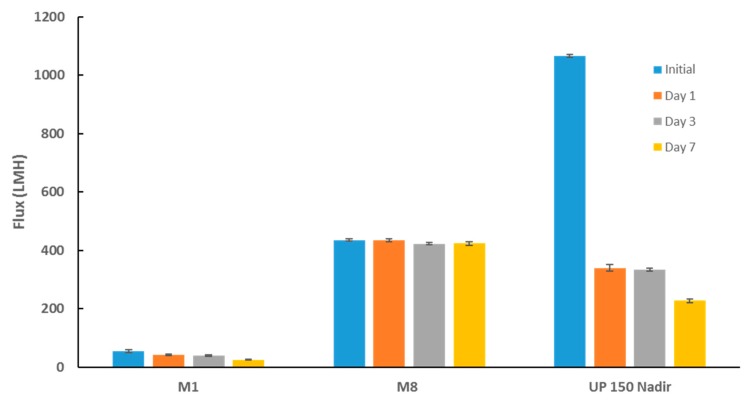
Membrane flux before incubation with bacterial suspensions and post incubation at different time points. Each bar represents the mean ± SD of three independent readings.

Figure 11c shows the BSA rejection values for M1, M8, and UP150 Nadir commercial membrane before and after filtration with *E. coli* suspension. All three membranes showed an increase in BSA rejection, with the greatest increase shown by the neat PES membrane (M1). This could be due to the fact that M1 samples experienced the highest levels of fouling (as evidenced by Figure 11b) and biofouling (see Figure 12, Figure 13, Figure 14 and Figure 15). It would be logical therefore, to expect higher rejection levels with fouled membranes since membrane pore clogging and cake layer formation would have occurred. These processes have both been seen to increase rejection of molecules since their passage is hindered with the obstruction of pore structures and in some cases decreasing the diameter of the pores [64,65,66]. In line with these deductions, M8 samples, which showed the highest biofouling resistance (see Figure 12, Figure 13, Figure 14 and Figure 15) showed the smallest increase in BSA rejection (2.3% increase). Despite the commercial UP150 Nadir membrane samples exhibiting weak biofouling resistance (see Figure 13, Figure 14 and Figure 15), BSA rejection only increased by a meager 3.6% after filtration with *E. coli* suspension. It should be noted however, the commercial UP150 Nadir membrane samples displayed the highest pristine rejection values (before fouling tests) (Figure 11a).

Overnight incubation of the fabricated membranes with Gram-negative and Gram-positive models (*E. coli* and *B. subtilis* suspensions respectively) elicited very different results for the neat and composite membranes as revealed by FESEM images in Figure 12. FESEM images for the membrane surfaces incorporating OMWCNT and AG showed a marked decrease and in some cases a total clearance (M8 samples incubated with *B. subtilis*) of bacterial colonization. The inhibitory effect on bacteria seen in these composite membranes can be explained by the membrane surface properties as already discussed. Firstly, the zeta potential values, which indicate strongly negative surface charges for the OMWCNT membrane samples, and the exceedingly hydrophilic nature of the membrane surfaces inferred from water contact angle measurements, render the synthesized composite membranes as possessing anti-adhesive membrane surfaces. Together these two key surface features of low-biofouling membranes cause the repulsion of negatively charged bacteria from the membrane surface, impeding subsequent attachment and colony formation. Another factor to consider is the membrane surface roughness. The AFM data shown in Figure 8 revealed that the composite membranes possessed significantly smoother surfaces, thereby reducing the surface area of bacterial colony attachment and multiplication.

In contrast, the neat PES membrane samples painted a different picture. FESEM images showed a carpet-like morphology where the entire membrane sample surface was covered by bacterial colonies (for both models tested). In light of the above discussion, this would seem logical, since the neat PES membrane possessed the least negative surface charge, lowest wettability, and roughest surface morphology, essentially bearing none of the hallmarks of low biofouling membranes.

The inhibition of bacterial colonization may also be further explained by the addition of AG, whose antibacterial nature has been alluded to in several studies [44,67,68]. Despite the exact mechanism behind AG’s antimicrobial nature remaining elusive, hypotheses have been put forward where the presence of antimicrobial enzymes [67] or high salt content [68] were postulated as being implicating factors. Nonetheless, it is reasonable to assume that the very hydrophilic nature of AG contributed in an additive manner to the role of OMWCNT in creating hydrophilic membrane surfaces.

Subsequently, anti-biofouling properties of the synthesized PES membranes alongside the two commercial membranes were evaluated. As described in the Methods section, membranes were treated with *E. coli* suspension covering their active surfaces and incubated overnight at 37 °C. Figure 13a shows the initial DW flux prior incubation followed by the DW flux for two consecutive 20 min filtration cycles after overnight incubation. Whilst all membranes exhibited a decline in flux after bacterial incubation, M8 samples exhibited the greatest flux recovery as depicted in Figure 13b. Importantly, the normalized flux for M8 samples not only surpassed the neat PES membrane samples but also outperformed the normalized flux for both commercial UP150 Nadir and UE50 TriSep samples (2-fold and 2.5-fold increase, respectively).

Flux performance during filtration of *E. coli* bacterial suspensions over prolonged periods yielded interesting differences between the composite and neat PES membrane samples. Whilst M8 membrane samples presented only a slight decline in flux over the entire filtration period (Figure 14a), M1 samples underwent a more prominent gradual decline in flux. Importantly, the commercial UE50 TriSep membrane samples fared worst in comparison to the neat and composite membrane samples, where a more pronounced flux decline was seen towards the latter half of the filtration test. The difference in flux stability was emphasized in the normalized flux data displayed in Figure 14b, where M8 samples had a normalized flux almost double that of M1, and a 24% increase from its commercial counterpart. In a similar experiment where the bacterial suspension was replaced with real locally obtained TSE, a similar trend was observed. M8 samples maintained a more stable flux throughout the filtration period, possessing a significantly higher normalized flux than both the neat PES and commercial membrane samples (Figure S2).

The biofouling resistance of selected PES membranes was further evaluated alongside commercial UP 150 Nadir membrane samples after incubation with *E. coli* suspension at incremental incubation times (Figure 15). Despite the commercial Nadir membrane exhibiting superior flux reading prior to incubation with bacterial suspension (more than twofold the flux for M8 samples), it experienced the worst biofouling as inferred from the sharp decline in membrane flux as a function of time. Moreover, the biggest decline in flux was seen at day 1 post-incubation where the membrane’s flux plummeted by almost 70%. This indicates that the membrane had already succumbed to biofouling processes within a space of 24 h incubation with bacterial suspension. Conversely, PES/OMWCNT membrane samples (as seen by M8 samples) retained an almost constant flux from initial flux readings taken prior incubation with bacterial suspension to final flux readings taken at day 7. The same experimental set up was repeated, using locally sourced real TSE in place of the *E. coli* suspensions. Similar results to those seen in Figure 15 were obtained where M8 samples exhibited superior biofouling resistance when compared to neat and commercial Nadir membrane samples (see Figure S3).

The results of biofouling tests shown in Figure 12, Figure 13, Figure 14 and Figure 15 can be explained in light of the contact angle, zeta potential, and AFM data collectively. As already discussed, the fabricated PES composite membranes containing OMWCNT and AG were shown to possess highly hydrophilic membrane surfaces, which exhibited larger negative charge, and smoother surfaces compared to neat PES membranes. These features are of paramount importance when designing membranes to tackle biofouling [8,26]. The smoother, negatively charged hydrophilic surfaces seem to disallow the attachment and adhesion of bacterial colonies onto its surface, rendering themselves anti-adhesive membranes for bacterial colonization.

**Figure 16:**
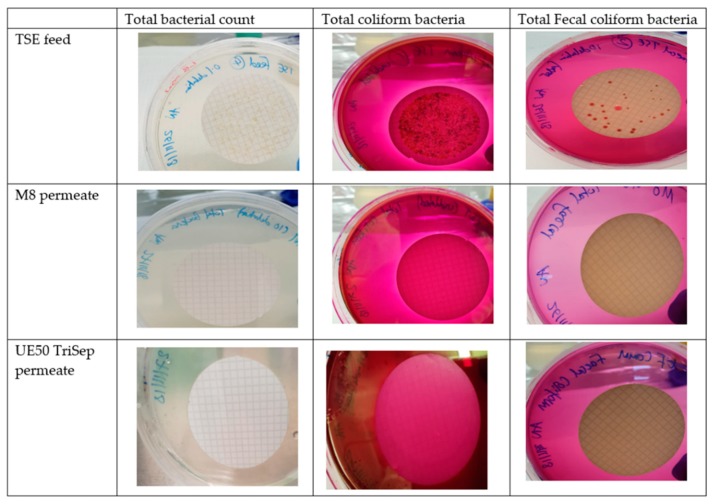
Images of agar plates with cellulose discs used for filtering either TSE feed or permeates of TSE filtration, using M8 and commercial UE50 TriSep membranes.

The presence of bacteria in TSE comes as no surprise, owing to the use of microorganisms in different types of sewage treatment processes such as activated sludge for instance [4]. Figure 16 displays the results for testing the bacterial content in feed TSE samples and permeates resulting from TSE filtration using M8 and the commercial TriSep UF membranes. Interestingly, none of the cellulose discs used for filtering the permeates of the composite PES and commercial membranes revealed any bacterial colonies for all three bacterial tests (total bacterial count, total coliform bacteria and fecal coliforms). These results indicate that both permeates were bacteria-free, unlike the TSE feed sample where an abundance of bacterial growth for all three bacterial tests was seen on its discs.

## 4. Conclusions

Characterization of OMWCNT confirmed successful oxidation of MWCNT starting material as evidenced by the increased oxygen content seen in the EDX mapping data and indirectly inferred from the TGA thermograms. Membranes incorporating both OMWCNT and AG consistently outperformed their neat PES counterparts when characterized for different parameters. Importantly the fabricated composite membranes exhibited lower surface roughness as evidenced by AFM data and a more negatively charged and hydrophilic membrane surface, as deducted from the zeta potential and water contact angle data.

Novel PES composite membranes showed superior performance when compared against neat PES or commercial UF membranes. Compared to neat PES, the hybrid membranes presented higher DW flux readings and fared significantly better than commercial membranes in terms of normalized flux in a range of fouling and antibiofouling tests. PES/OMWCNT membranes showed that they are able to retain a stable flux during prolonged filtration of both bacterial suspensions and real TSE.

PES/OMWCNT membranes also indicated better resistance to organic fouling as modeled by filtration of BSA solutions, with a marked decrease in irreversible fouling seen across all hybrid membranes. Importantly, the prepared membranes containing OMWCNT and AG exhibited anti-adhesive properties against both Gram-negative (*E. coli*) and Gram-positive (*B. subtilis*) bacterial models employed. Furthermore, these membranes were successful in eliminating the bacterial species from TSE during filtration. These enhanced performance indicators can be attributed to the increased negative surface charge, surface smoothness, and increased hydrophilicity possessed by the hybrid membranes. Together these attractive features of low-biofouling membranes hinder bacterial attachment to membrane surfaces, thereby limiting biofouling processes.

## Figures and Tables

**Table 1:** Composition of cast polyethersulfone (PES) membranes. M1 is a neat PES membrane. N,N-dimethylacetamide (DMA) solvent was used in the preparation of all membranes.

Membrane	PES (wt. %)	AG (Arabic Gum) (wt. %)	OMWCNT (Oxidized Multiwalled Carbon Nanotubes (wt. %)
M1	16	-	-
M2	16	1	-
M3	16	1	0.1
M4	16	1	0.3
M5	16	1	0.5
M6	16	1	1
M7	16	1	3
M8	16	1	5
M9	16	-	0.3

**Table 2:** Energy dispersive X-ray spectroscopy (EDX) mapping data for multiwalled carbon nanotubes (MWCNT).

Element	Series	[wt. %]	[norm. wt. %]	[norm. at. %]
Carbon	K-series	90.1	90.1	92.9
Oxygen	K-series	8.4	8.4	6.5
Iron	K-series	0.7	0.7	0.5
	Sum:	100.0	100.0	100.0

**Table 3:** Energy dispersive X-ray spectroscopy (EDX) mapping data for oxidized multiwalled carbon nanotubes (OMWCNT).

Element	Series	[wt. %]	[norm. wt. %]	[norm. at. %]
Carbon	K-series	75.9	75.9	81.0
Oxygen	K-series	23.5	23.5	18.9
Iron	K-series	0.2	0.2	0.1
	Sum:	100.0	100.0	100.0

## Supplementary Materials

The following are available online at https://www.mdpi.com/2077-0375/9/2/32/s1, Figure S1. EDX mapping for MWCNT (**a**) and OMWCNT (**b**); Figure S2. Graph showing flux readings for filtration of real TSE with selected fabricated membranes and the UF commercial membrane (**a**) and the normalized fluxes for these membranes after TSE filtration (**b**); Figure S3. Membrane fluxes before and during incubation with real TSE at different time points.
