# Supplementary material for: Antibiofouling Performance by Polyethersulfone Membranes Cast with Oxidized Multiwalled Carbon Nanotubes and Arabic Gum
Source: Membranes (Basel). 2019 Feb 22;9(2):32. doi: 10.3390/membranes9020032 (PMC6410110; doi:10.3390/membranes9020032)
Supplement: Supplementary file 1 [file membranes-09-00032-s001.pdf]

membranes

Supplementary Materials: Antibiofouling performance by Polyethersulfone membranes cast with oxidized multiwalled carbon nanotubes and Arabic Gum

Firstname Lastname [1], Firstname Lastname [2] and Firstname Lastname [2,*]

Ahmad Najjar [1,*], Souhir Sabri [2], Rashad Al-Gaashani [2], Muataz Ali Atieh [2] and Viktor Kochkodan [2]

1 Hamad Bin Khalifa University HBKU, College of Life and Health Sciences, Qatar; ahmnajjar@hbku.edu.qa
2 Qatar Environment and Energy Research Institute (QEERI) Hamad Bin Khalifa University (HBKU). mhussien@hbku.edu.qa
* Correspondence: ahmnajjar@mail.hbku.edu.qa

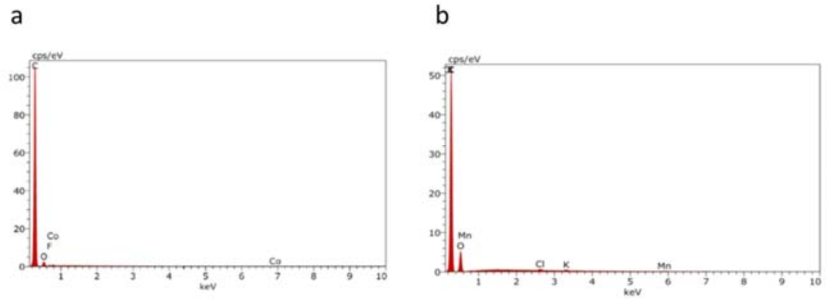

Figure S1. EDX mapping for MWCNT (**a**) and OMWCNT (**b**).

Commented [M1]: Please replace with a sharper image.

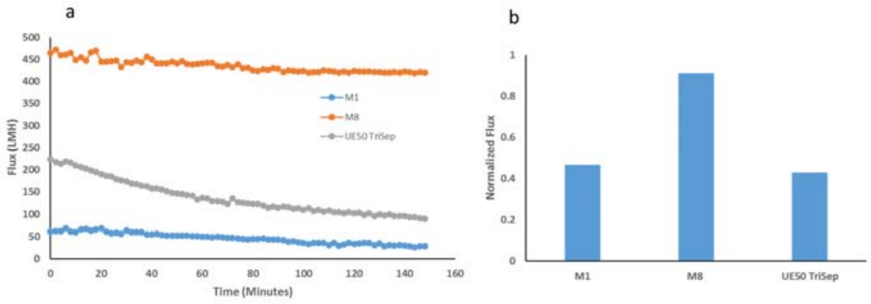

Figure S2. Flux readings for synthesized and UE50 TriSep membranes over a prolonged period of real TSE filtration (**a**). Normalized flux data for each of the three membrane samples (**b**).

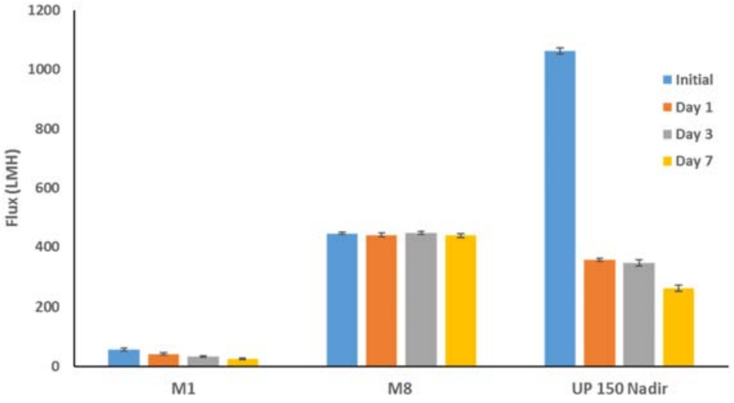

Figure S3. Membrane flux before incubation with real TSE and post incubation at different time points.

© 2019 by the authors. Submitted for possible open access publication under the terms and conditions of the Creative Commons Attribution (CC BY) license (http://creativecommons.org/licenses/by/4.0/).
